# General Practitioners’ Perceptions on Prescribing Coastal Visits for Mental Health in Flanders (Belgium)

**DOI:** 10.3390/healthcare13131599

**Published:** 2025-07-03

**Authors:** Alexander Hooyberg, Luka De Wever Van der Heyden, Marine I. Severin, Stefaan De Henauw, Gert Everaert

**Affiliations:** 1 Department of Public Health and Primary Care, Ghent University, Corneel Heymanslaan 10, 9000 Ghent, Belgium; 2 Department of Head and Skin, Ghent University, Corneel Heymanslaan 10, 9000 Ghent, Belgium; 3 Flanders Marine Institute (VLIZ), Jacobsenstraat 1, 8400 Ostend, Belgium; 4 Department of Experimental Clinical and Health Psychology, Ghent University, Henri Dunantlaan 2, 9000 Ghent, Belgium; 5 Centre for the Psychology of Learning and Experimental Psychopathology, KU Leuven, Tiensestraat 102, Box 3712, 3000 Leuven, Belgium

**Keywords:** Blue Care, nature, general practitioners, attitude, barriers to treatment, mental health

## Abstract

Background: Increasing evidence suggests that visiting the coast benefits mental health and that coastal prescribing is a promising societal endpoint. General practitioners (GPs) are the pivotal access point for patients to receive diagnosis and treatment, but little is known about their perspective on recommending patients to visit the coast. Methods: This study applied qualitative semi-structured interviews to explore GPs’ perspectives on coastal prescribing in Flanders. We interviewed eleven GPs (aged 32–69 years) and inspected their responses using inductive thematic analysis. Results: Results show that the interviewed GPs generally believed in the therapeutic benefits of the coast, but also acknowledged risks associated with crowding and patient-specific effects. Six barriers were identified for coastal prescribing: feasibility concerns, lack of awareness, prioritizing physical exercise or visiting nearby green nature, anticipating low motivation of the patient, feeling pressure to prescribe medication, and needing more scientific evidence. As solutions, they proposed gathering more scientific evidence and raising awareness. Finally, the GPs regarded their field expertise as valuable in helping to recruit patients for follow-up research on the health effects of the coast. Conclusions: Our findings highlight the importance of engaging GPs, patients, and other stakeholders to identify key knowledge gaps before co-creating coastal prescribing in healthcare.

## 1. Introduction

Approximately 13% of the global population experiences psychological distress, and the prevalence of mental health disorders is on the rise [1]. Optimal mental health is crucial for daily functioning, the maintenance of healthy interpersonal relationships, and the ability to cope with individual, structural, and community-level risk factors [2]. The growing incidence of mental health problems appears to be influenced by a combination of individual and societal factors. Contemporary lifestyles, consequences of the COVID-19 pandemic, shifting cultural expectations, and political instabilities place considerable strain on both the brain and the body [3]. The World Health Organization currently endorses a range of therapeutic interventions, including psychosocial approaches such as cognitive behavioral therapy and the administration of psychotropic medications. While these interventions demonstrate efficacy, their benefits are often transient, leading to high rates of relapse [4,5]. Additionally, the stigma and potential discrimination associated with mental health treatment can discourage individuals from adhering to therapy [1].

To effectively address the ongoing mental health crisis, spending time in coastal and other natural outdoor environments has been proposed as one of the promising complementary therapies to treat psychological and somatic burdens [6,7,8,9]. Nature prescription programs are increasingly being implemented and have been referred to as ‘Nature Prescriptions’ [10,11], ‘Blue Care’ [12,13], or ‘Green/Blue (Social) Prescribing’ [14,15]. They have been implemented on various scales and have involved various methods, from single GPs prescribing visits to the coast to their patients on an individual basis, to national nature-on-prescription systems streamlining referral to nature providers via community workers or link workers. ‘Coastal prescribing’ specifically refers to healthcare providers recommending their patients to visit seaside destinations with assumed health benefits (e.g., beaches, dunes, perhaps also dikes and seaside resorts). Coastal prescribing seems to be especially promising, because there is increasing evidence for the mitigative, restorative, and instorative effects of coastal blue spaces [16,17,18] and their superiority compared to inland blue and green spaces [19]. Despite the growing societal and scientific enthusiasm for nature-based interventions, and for interventions at the seaside in particular [13], few studies have investigated the perspective of healthcare providers, such as general practitioners (GPs), on these interventions [20,21,22,23,24].

Understanding the perspective of GPs towards coastal prescribing is valuable for researchers for two reasons. First, GPs can identify knowledge gaps, practical considerations, and challenges for research to address to enable the implementation of coastal prescribing, such as how effective coastal prescribing would be for different patient profiles, what potential barriers and facilitators may motivate GPs and patients to adhere to coastal prescriptions, and the overall feasibility within healthcare settings. Second, the next step in fundamental research is to investigate how exposure to the coast can restore malfunctioning processes in patients with various emotional, cognitive, and psychosomatic burdens. To recruit these patients, GPs and other healthcare providers (e.g., psychologists, psychiatrics, physiotherapists) are crucial for appropriate pre-selection and referral to ongoing clinical trials.

This study aimed to capture the perspectives of GPs in Belgium with respect to prescribing coastal visits. More specifically, using a qualitative approach with semi-structured interviews, this study explored GPs’ attitudes (i.e., previous coastal/green prescriptions they might have given, opinions and knowledge about the efficiency of coastal prescribing on health, and the most suitable patient profiles), the practical and societal barriers and opportunities they identify for implementing coastal prescribing, and their opinions on being involved in further research.

## 2. Materials and Methods

### 2.1. Semi-Structured Interviews

Semi-structured interviews follow a list of predefined questions in an interview guide to gather consistent and relevant information from all respondents, while allowing the interviewer to deviate from the questions to explore the diversity and depth of the interviewee’s opinions, experiences, perceptions, knowledge, and behaviors [25,26,27,28]. In this study, the interview guide consisted of four parts (see Supplementary Material S1). The first part asked for the GP’s age, the postal code of their practice, the years active as a GP, and their personal relationship to the coast. The second part focused on their attitudes towards prescribing coastal visits, i.e., whether and how they prescribed a coastal visit (or nature visit) in their practice, if they knew colleagues who did, what they thought about the efficiency of coastal visits for treating mental vulnerabilities, and what patient profiles they deemed most suitable for receiving coastal prescriptions. The third section explored the practical and societal barriers they could identify for implementing coastal prescriptions in their practice, and the possibilities they could identify to lift those barriers. The final part of the interview guide assessed the extent to which GPs wanted to be involved in future research on the health effects of the coast.

We conducted a prior literature review to formulate the main questions in the guide, to enhance the reliability and validity, and to prepare adequate follow-up questions. The review included Dutch- and English-language academic sources (peer-reviewed journal articles, preprints, books, book chapters, theses, and dissertations) in PubMed and Web of Science. We also included known master’s theses about green social prescribing in the database of master’s theses of the Belgian Interuniversity Collaboration for General Practitioner Training (https://www.icho-info.be/application/content/thesislist (accessed on 1 February 2024)). The search terms included (attitude) OR (outlook) OR (perception) AND (general practitioner) OR (caregiver) OR (health provider) AND (blue space) OR (green space) OR (exercise) AND (prescription). Ultimately, five studies served as inspiration for the interview guide [20,21,22,23,24].

The usability and relevance of the interview guide was further improved by pilot-testing it with two GPs. This involved evaluating whether the guide was clear, logically organized, and efficient for collecting the necessary information. The pilot tests mainly led to refinements of the interview guide. For example, a question about participants’ attitudes towards other alternative therapies (e.g., mindfulness) was removed as it caused confusion and diverged too far from the main objectives. The original Dutch version of the interview guide and English translation are available in the Supplementary Material S1.

The interviews were held either in person or online, depending on the participants’ preference, and lasted between 20 and 90 min. The audio of all interviews was recorded and transcribed afterwards.

### 2.2. Recruitment and Sample

GPs were recruited via social media (Instagram and Facebook); local GP networks, the local umbrella organization Domus Medica; the Local Health Authorities in Bruges, Ostend, Antwerp, and Ghent; and personal contacts. The recruitment letter and advertisements mentioned the relevance, purpose, and methodology of the study, and as such focused on GPs with a relatively strong interest in favor or against coastal prescribing. The principle of saturation was used to determine sample size, meaning that data collection was considered complete if no new categories or themes emerged after two to three consecutive interviews [29]. Eleven GPs were interviewed because no new information arose after eight interviews.

Of the eleven GPs interviewed, two had their practice at the Belgian coast, one in the only coastal province of West Flanders but not at the coast, two in the province of East Flanders, five in the province of Antwerp, and one in the province of Hainaut. Their age varied from 30 to 70 years old (M = 49 years, SD = 13 years; one GP was recently retired). They were active as GPs for 3 to 42 years (M = 21 years, SD = 15 years).

### 2.3. Data Analysis

We used an inductive thematic analysis to extract the various perspectives that GPs have about coastal prescribing. Inductive thematic analyses involve six steps [30]: (1) familiarization with the responses, (2) labeling relevant phrases in the responses with codes (e.g., ‘barrier—distance to the coast’), (3) collating the coded phrases into potential themes (e.g., ‘barrier: feasibility’), (4) reviewing these themes in relation to the coded phrases, (5) defining and naming the final themes, and (6) producing a report with relevant excerpts. The analysis was conducted using the Nvivo11 software program.

## 3. Results

All quotes from the GPs reported in the following sections were translated from Dutch to English.

### 3.1. Attitudes Towards Coastal Prescriptions

#### 3.1.1. GPs’ (Coastal) Prescribing Behavior

Two of the eleven GPs regularly recommended visits to coastal environments. One of these GPs lived by the coast. Eight GPs were aware of colleagues who recommended green nature visits and did so themselves. Three GPs reported that they did not recommend coastal or green visits and did not know colleagues who did.

#### 3.1.2. Opinions About Effects of the Coast on Health

All GPs believed that visiting the coast would have a relaxing effect and would enhance the overall mental health and well-being of patients, but this attitude may have originated from mentioning these effects during the recruitment and introduction to the interview. Seven GPs further believed that the coast encouraged physical activity. Seven GPs noted that the air at the coast was healthier, which they believed also promoted physical health. Finally, five GPs stated that the effects of the coast were similar to those of green nature, and two GPs thought that coastal and inland blue nature might have better health effects than green nature. Participant 8 illustrated this point: “Many people just go to the beach during their lunch break, get some fresh air, and then return. It helps to clear your mind, the fresh air, the sea air. So, I strongly believe in its relaxing and well-being effects” (24 April 2024). Participant 10 said, “I think the sea itself can have a different or greater impact than green nature (…) because for me, walking by the sea feels quite different than walking in a park. So, I think it can definitely have added value” (15 May 2024).

The GPs also described some potential negative effects of the coast on health. Four GPs pointed out that the coast can be too crowded in summer to experience its relaxing effects, and one GP mentioned the risk of injuries.

#### 3.1.3. Relevant Patient Profiles

Several patient profiles were specifically mentioned during the interviews. Seven GPs reported that they would be more likely to refer patients to the coast who were dealing with depression or anxiety-related, psychosomatic, or stress-related issues such as burnout. In the words of Participant 8, “If we’re talking about burnout, depression… I would specifically recommend the coast. I feel that the openness and expansiveness are mentally better than a city or even a forest that is quite dark and solitary” (24 April 2024). Four GPs indicated that their recommendation would depend on the severity of the patient’s symptoms when it came to anxiety or depression-related issues. For patients with very severe symptoms, the motivation threshold to visit the coast would be too high, and they would instead focus on a low-threshold treatment such as a ten-minute walk outside. As Participant 3 noted, “People who are clinically depressed usually have no motivation for anything, and I already have a hard time getting them to come to me every few weeks” (27 March 2024).

The interviewed GPs raised mixed issues with regard to loneliness. Three GPs reported that they would be less inclined to refer patients with loneliness issues to the coast, as this might not alleviate the feeling of loneliness or could even worsen it. However, two GPs argued that the coastal region could be a low-threshold environment for lonely individuals to engage in activities. Participant 5 argued that “There are walkers who are out there alone, whether they are blowing off steam or jogging, so it’s not noticeable. I think it’s a bigger barrier to go to a restaurant alone than to walk alone on the coast” (17 April 2024).

### 3.2. Barriers for Implementing Coastal Prescriptions

#### 3.2.1. Feasibility for the Patient

The greatest barrier identified in all interviews was feasibility for the patient. This included financial aspects, time constraints, and the distance to the coast. This barrier was less significant for the two GPs who lived by the coast. In the words of Participant 8, who had her practice at the coast, “It shouldn’t be a burden to reach the coast because, if you’re prescribing it, it means, (…) you need it, there’s something that would justify the prescription, and it shouldn’t be an additional burden or stress to fulfill that prescription” (24 April 2024). The GPs emphasized that for different patient profiles (single-parent families, mentally or physically disabled individuals, people with a low socio-economic status, etc.), it would often be unfeasible to carry out a coastal visit on referral. Four GPs additionally reported the risk of widening the health inequality gap. Participant 5 highlighted, “But we need to ensure that we’re not creating an elite stream of coastal-visiting patients and a stream that can barely afford it” (17 April 2024).

#### 3.2.2. Lack of Awareness

A major barrier that eight GPs identified was simply not considering the possibility of coastal prescription. Notably, one of these GPs lived by the coast. As Participant 3 explained, “Your question is a bit like why don’t you prescribe that medicine, but I actually didn’t know that medicine existed. So, it’s not yet in my routine, it’s not in my book of pills, even though it could be there” (27 March 2024).

#### 3.2.3. Priority for (Green) Exercise

According to five GPs, encouraging physical activity was a higher priority than recommending a coastal visit, as it would cause better health effects and was more accessible for the patient. For this reason, visiting the coast would also be less likely to be mentioned in the GP’s practice, as the focus is on promoting physical activity. They thought that exercising in nature would bring more health benefits than in urban environments, but whether this nature was blue or green was considered of little importance. Participant 8 explained, “If you live in a forest, go to the forest. If there is a park nearby, go to the park. So, I think the focus on physical activity is the most important” (24 April 2024).

#### 3.2.4. Personal Preference and Patient Motivation

For five GPs, personal preference and patient motivation emerged as barriers to prescribing a coastal visit. The health effects of a coastal visit were considered to be dependent on individual factors. Patients who are open to a coastal visit, have had previous positive experiences with the coast, have positive perceptions of the coast, or do not experience too much stress in reaching the coast were found to be the most likely to receive benefits from coastal visits. Patients for whom this is not the case were thought to experience fewer or no benefits. Participant 8 emphasized, “I maintain that it is very personal. For one person, the coast might be better, for another a forest, and for another, going abroad. They don’t necessarily need to be at our Belgian coast. So, I think everything in healthcare is personal” (24 April 2024).

#### 3.2.5. Pressure to Prescribe Medication

Four GPs felt the pressure to prescribe medication over a visit to the coast. On the one hand, they thought that patients expected medication, and on the other hand, they thought that patients might feel they are not being taken seriously if a coastal visit is recommended as an alternative to medication. Participant 3 emphasized, “And it’s very difficult to say, ‘I don’t want to give you a pill’, because the expectation is there” (27 March 2024). Participant 4 also mentioned, “So there’s already an assumption that the patient won’t be happy with this [a coastal visit]. While it might turn out to be fine, we need to try to present it and see what the patient’s reaction is” (29 March 2024). Nevertheless, all GPs agreed that a coastal visit did not necessarily have to be accompanied by medication. Three GPs also mentioned that they did not feel pressured to prescribe medication since exploring psychosocial issues in a non-medicalizing way was normal within their practice, and normalizing this approach might make patients more open to it.

#### 3.2.6. Need for More Evidence

Five GPs would require more evidence on the fundamental health effects of coastal visits on an individual. However, they emphasized that this was not the primary reason they did not currently recommend coastal visits, but additional evidence would provide a solid basis for their recommendation. Four GPs did not need additional evidence. They were sufficiently convinced of the health effects of the coast (which may in part be caused by mentioning the health benefits of the coast in our recruitment letter).

### 3.3. Potential Opportunities

#### 3.3.1. Developing a Broader Framework

Four GPs believed that coastal prescribing might better fit within a broader framework, such as the framework ‘Exercise on Prescription’ that already exists in Flanders and is ran by the Flanders Institute for Healthy Living (in Dutch: Vlaams Instituut Gezond Leven; www.gezondleven.be/projecten/bewegen-op-verwijzing (accessed on 1 July 2025)). Participant 7 explained that “It seems more logical to me, for example, to make coaches that already do ‘exercise on referral’ aware that people could also go to the coast, rather than having a separate coastal coach” (24 April 2024).

Five GPs were reluctant towards developing a framework for organizing coastal visits, because they argued it was not a priority to use resources from health insurance funds or the regional government to implement a referral for coastal visits, as they believed alternative prescriptions (e.g., visiting a park, physical activity) could provide similar health benefits. Participant 8 noted, “Is it a priority to now invest money from the health insurance fund in this? Yes, I am even hesitant about that. Because I think general exercise already has a lot of evidence and added value. If we can stimulate everyone to move in their own environment and do that, it would be of greater benefit than a coastal visit” (24 April 2024). Four GPs mentioned that they would need more scientific evidence about the difference in health effects between the coast and green nature, as a referral to the coast involves more barriers than green nature for GPs who do not live near the coast. If a coastal visit offered significantly more (or different) health benefits than visiting green nature, they would consider allocating resources to implement referrals for coastal visits. Participant 11 said, “Yes, there must be a substantial difference [between the health effects of green nature and the coast]. Then you could say, if the forest improves by 40 percent, but the coast maybe by 60 percent (…) Otherwise, I would only recommend it for people living in West Flanders or those living near the coast” (18 May 2024).

#### 3.3.2. Interdisciplinary Awareness

Eight GPs reported that increased interdisciplinary collaboration amongst various healthcare providers (including GPs, psychologists, and physiotherapists) could be beneficial for implementing a coastal visit on referral. By this, they meant increasing awareness among healthcare providers about the benefits of a coastal visit and the opportunity to recommend it to their patients. According to the GPs, increasing awareness could also motivate patients to visit the coast. Participant 1 emphasized, “When I see someone with acute lower back pain and I say, ‘Look, I can give you a pill, but it won’t help much,’ what does help is movement or swimming. And if the same people then go to the emergency room, they come out with a bag of pills. Everyone says something different and the recommendations are unclear” (26 March 2024).

Seven GPs highlighted the role of organizations such as health insurance funds or GP circles in raising awareness about both the health effects of coastal visits and the possibilities for making recommendations. This could be achieved, for example, through (media) campaigns. Participant 5 stated, “If this were emphasized more in a campaign, it would help (…) to make it more evident and also to make the patient realize it. When the ‘Exercise on Prescription’ program started, it was also helpful that there were brochures and a bit of a campaign around it” (17 April 2024).

#### 3.3.3. Group Activities/Buddy System

Six GPs proposed recommending coastal visits in the form of organized group activities to include all patient profiles. This could also be achieved through a ‘buddy system’, where specific patient profiles are accompanied by a helper (the buddy) supporting the patient with organization, transportation, or arrival at the coast. Some GPs mentioned the need for reimbursement of train tickets to the coast for patient groups needing financial support. However, five GPs believed that, despite organizing group activities or a buddy system, not all patient populations would be reached. It might even be a barrier for some patients with mental disorders that are associated with symptoms of social withdrawal (e.g., burnout, anxiety disorders). Participant 2 emphasized that “The people you attract with an offer from a practice or from the health insurance fund or whatever are people who are already actively looking around. The people who are struggling and really need it are isolating themselves and need to be approached to do something like this” (26 March 2024).

### 3.4. Involvement of GPs in Research

According to eight GPs, it is important to involve them in future research on the fundamental effects of the coast on (mental) health, on the one hand because the implementation needs to be adapted to the context of the GP, and on the other hand because GPs are experts on the patient’s context. Their role in future research would primarily be to recruit patients, as they can select which patients would benefit most from a coastal visit. In the words of Participant 10, “Yes, recruiting patients, referring them (…) that can definitely be our role. Interpreting the results, I don’t think I’m well-versed in that. I think that is better for other people. But picking and selecting patients, determining who is a good candidate, that we can certainly do” (15 May 2024). Four GPs also emphasized that their involvement in research should involve as little administrative work as possible, as otherwise, GPs might not be motivated to participate.

## 4. Discussion

### 4.1. Moving Towards Coastal Prescribing

This study demonstrated that the attitude of GPs towards prescribing coastal visits to their patients was predominantly supportive. All interviewed GPs believed that spending time at the coast has beneficial effects on mental health. Although they may have been primed by the recruitment letter and briefing at the onset of the interviews mentioning that the coast provides health benefits, the GPs attributed the health-promoting effects of the coast primarily to its relaxing benefits, improved air quality, and the encouragement to engage in physical activity. These factors are also highlighted in previous studies as potential key mechanisms explaining the relationship between the coast and health [16,18,31,32,33,34]. Several GPs warned that these benefits would diminish in summer when there is crowding, which is in line with findings from previous research showing that social contact is beneficial as long as the feeling of safety is preserved [35,36]. The High Level Panel for a Sustainable Ocean Economy [37] and the World Economic Forum [38] also advise limiting periods of overcrowding to make coastal tourism more sustainable.

The GPs considered the coast to be particularly effective for patients with depression, anxiety, psychosomatic, or other stress-related complaints, though this also depended on the severity of the symptoms, their socio-demographic background, and their residential proximity to the coast. This aligns with earlier research suggesting that interaction with the coast has beneficial effects on the treatment of mental health conditions such as depression and anxiety disorders [13], but that the effects can be influenced by the nature and severity of a patient’s symptoms [39,40]. For example, women with suicidal tendencies may experience worse symptoms in the presence of the coast [41]. These findings underscore the importance of investigating which specific patient profiles would benefit most from a coastal visit and which would benefit less.

Six barriers were identified by the GPs that prevented them from (more frequently) recommending a coastal visit in their practice. The barrier most often emphasized was the feasibility of a coastal visit due to financial reasons, lack of time, and the distance many patients would need to travel. In a previous qualitative study on GPs’ perspectives on green prescriptions, this feasibility aspect was also prominent [20]. Interestingly, the interviewed GPs in the current study did not explicitly mention feasibility issues for green prescriptions when compared to coastal prescriptions, suggesting that GPs have stronger feasibility concerns for coastal vs. green prescriptions. This may be attributed to the smaller distance to nearby green spaces compared to the coast in Belgium, and hints to a perception that the quality of green spaces is of little importance and that suitable green spaces are omnipresent in Belgium.

Several GPs emphasized that the coast is less accessible for patients with lower socio-economic status (SES), potentially resulting in less adherence of this group to coast-based interventions. This is supported by various studies indicating that patients’ SES influences the relationship between natural environments and health. More specifically, individuals with lower SES are more likely to have less access to nature but may derive more health benefits from natural environments [42,43,44,45]. However, the moderating role of SES was recently disputed by an 18-country cross-sectional analysis [46]. Furthermore, research shows that failing to reach low-SES groups when implementing nature-based interventions can exacerbate the gap in health inequality [43,47]. Thus, the feasibility of coastal prescriptions is especially a great concern for lower socio-economic groups, and organizational solutions will have to be inclusive enough to overcome this barrier for all patients.

The GPs in the sample reported five other barriers to recommending a coastal visit: not thinking of it, anticipating low patient motivation, needing more evidence, prioritizing exercise, and pressure to prescribe medication. The first three of these barriers largely align with a previous study [20] on GPs’ perspectives on green prescribing (i.e., not thinking of it, anticipating low patient motivation, and the need for more evidence). Three barriers (feasibility, anticipating low patient motivation, and pressure to prescribe medication) were related to the patients’ perspective, which suggests that GPs’ decision-making is influenced by patients’ opinions and underscores the need to involve patients in further research on coastal-based interventions. The GPs also felt pressure to prescribe medication, because the patient would expect to receive medication. GPs are often in a difficult position needing to consider patients’ situations and motivations alongside the effectiveness of existing therapies and the recommendations of other healthcare providers (e.g., specialists), while still having a personal view on what would be best for the patient [48,49,50,51,52,53,54,55,56]. Given the barriers identified by the GPs in this study, it seems that there are still many uncertainties among GPs and patients with regard to the therapeutic potential of the coast in comparison to green environments and medication and how the wider healthcare system would support coastal prescribing clinically and practically.

Four of the interviewed GPs were open to a coastal prescribing framework similar to, or part of, ‘Exercise on Prescription’ to overcome the aforementioned barriers. This was also observed in the previous study [20] on green nature. ‘Exercise on Prescription’ is a program that raises awareness about the value of physical exercise for health and involves ‘exercise coaches’ that support patients in starting a more active lifestyle—small steps at a time—through a referral letter from a GP. Patients receive points with which they can receive free individual or group counselling from an exercise coach. Several GPs proposed integrating green/coastal visits into such a broader framework, in which the exercise coaches would propose the opportunities for physical activity in coastal/green nature in the patient’s region of interest while considering potential feasibility barriers. The GPs identified three components that should be part of a coastal prescribing framework: a buddy system or group activities, campaigns to raise awareness of the health benefits of the coast, and interdisciplinary collaboration. Establishing a group/buddy system appears to be a feasible option for organizing coastal visits in general practice, as several studies have already organized nature-based interventions in groups, resulting in positive health outcomes and increased social contact [57,58,59,60]. However, GPs emphasized the risk of excluding certain patient groups (e.g., lonely patients, patients with depression or anxiety-related complaints that tend to socially isolate themselves) in organizing group activities, as group activities may be a barrier for these patients. Various studies also show that the treatment preferences of patients with depression or anxiety-related complaints often favor individual guidance over group treatments [61,62]. These findings highlight the need for a tailored approach. Depending on the envisioned effectiveness of the coastal treatment and patients’ preferences and the severity of their symptoms, GPs might adopt different communication strategies (e.g., directive, suggestive) to help guide the patient to follow the best (coastal) therapy in their own way [52].

Awareness campaigns could inform GPs, other healthcare providers, and patients about the existing knowledge on the health benefits of the coast and foster interdisciplinarity. In the ‘Exercise on Prescription’ program, now almost ten years in the running, the biggest challenge remains motivating GPs to prescribe physical exercise. Despite the enormous evidence showing the strong effects of physical activity on health and well-being and the repeated recommendations for prescribing this to patients [63,64,65,66], this does not seem enough to convince many GPs to prescribe exercise. Meanwhile, patients have their own expectations, values, and priorities in life, which may hamper their ability to devote time and money to particular treatments. Acknowledging GPs’ unique position and experience with patients’ motivations and adherence to different kind of therapies, perhaps awareness campaigns should be mainly targeted towards informing citizens of the value of outdoor (coastal) activities for maintaining and restoring health and well-being. Thus, not only is required further research on the effects of the coast on (mental) health, but also on the efficiency of different communication strategies in awareness campaigns and between GPs and patients.

However, five out of the eleven GPs did not believe that financial resources from the government or health insurance funds should be allocated to the organization of a coastal prescribing framework. They emphasized that focusing on physical exercise was more important and that they would most likely recommend any type of nature, and not the coast specifically. Nevertheless, the reluctance of GPs to invest resources in coastal visits might be reconsidered in light of potential cost savings. Several studies suggest that the effects of nature on health results reduce health burdens and healthcare costs [67,68]. Furthermore, every euro invested in prevention yields an estimated fourfold return by avoiding costs in curative care, such as sickness absenteeism [69,70,71,72]. These estimations suggest that if additional evidence confirms that coastal visits provide significant health benefits over green visits—which some of the interviewed GPs believed—and these added benefits are cost-effective for the patient, reconsideration of the investment in organizing a framework for coastal referrals may provide long-term clinical and economic benefits.

### 4.2. Limitations

This study captured the perspectives of only eleven GPs on coastal prescribing, which hampers the generalizability of the findings and the ability to formulate strong recommendations for policy. The small size of Belgium and its relatively uniform sandy beaches further limit the generalizability outside Belgian borders, and the relatively good access to the coast and general familiarity with the coast among the population are relatively distinct compared to neighboring countries. There was a potential selection bias, as GPs with a particular (dis-)interest in coastal prescribing were more likely to participate in the recruitment process. The recruitment letter clearly stated that the study was about the attitude of GPs towards recommending coastal visits. Also, only two GPs working in coastal areas were recruited and coastal GPs may be more inclined to prescribe coastal visits to their patients, but the small sample limits the ability the compare opinions between GPs located at the coast vs. inland. Nevertheless, to the authors’ knowledge, this is the first study that mapped healthcare professionals’ perspectives on coastal prescribing. The data contained a saturated and diverse set of attitudes, barriers, and needs of GPs, of which several were not previously described (e.g., priority for physical activity, differences between coastal and green prescribing). The study focused specifically on prescribing actual visits to the coast. Although being physically present at the coast is preferred to maximally transfer the benefits [73,74], it should be acknowledged that coastal prescribing may also entail virtual coastal interventions, such as documentaries about the ocean, sounds of waves, and mental envisioning of the coast. Lastly, the study did not investigate the environmental consequences of coastal prescriptions, such as ecological degradation. Coastal prescribing may cause additional overcrowding and pressure on ecologically stressed coastal areas, which are likely to result in negative impacts on the coastal experience, health, and well-being of all coastal visitors and residents.

### 4.3. Recommendations for Future Research

Now that we know the perspectives of eleven GPs, the next logical step is to capture the nation-wide variability in GPs’ perspectives. GPs repeatedly compared coastal with green prescribing when expressing their concerns with regard to feasibility, their believed effects of the coast, the patient specificity of these effects, the priority to recommend physical exercise, and an overarching framework as solution. Therefore, future research should disentangle in which situations GPs are more prone to providing coastal vs. green vs. medication prescriptions, and in which situations each type of exposure is most effective. Investigating the effects of coastal vs. green prescriptions is also useful from an empirical point of view. Regardless of the type of environment, going outdoors stimulates physical activity, social interactions, and stress relief, and researchers may either investigate the whole effects of coastal environments (e.g., the effects of walking at the beach compared to staying home), or the added value of coastal environments compared to inland environments (e.g., the effects of walking at the beach compared to walking in a forest and in the city). There is a relatively good understanding of the added benefit of green over urban environments [11,42,75,76,77,78], but there is much less evidence and understanding about the added benefit of natural and urban environments at the coast compared to inland green and urban environments [8,16,39,79]. The interviewed GPs also noted the pressure to prescribe medication, which suggests that future research comparing coastal prescribing with medication prescribing can reveal valuable information for GPs to choose the optimal treatment for their patients. From a practical point of view, future research should focus on the feasibility, cost-effectiveness, and implementation possibilities of coastal prescribing frameworks and the most effective communication strategies in awareness campaigns and in GPs’ practice. Finally, the GPs emphasized that patients’ perspectives are equally important, and future research should target patients’ reactions to receiving coastal prescriptions, including their perceptions about the effects of coastal visits as therapy for their mental health, their barriers, practical needs, and potential motivators.

## 5. Conclusions

This study aimed to explore GPs’ perspectives on referring patients to coastal visits to promote mental health. Eleven GPs were interviewed using semi-structured qualitative interviews. The results indicated that the GPs held a predominantly supportive attitude towards recommending a coastal visit. They believed that the coast contributes to patients’ mental health and could be effective as a (complementary) treatment for various patient profiles, but especially for patients with stress-related disorders. However, coastal prescribing was rarely implemented in practice. Six barriers emerged from this study: feasibility, lack of awareness, low priority, patient motivation and preference, pressure to prescribe medication, and a need for more evidence. The participants highlighted the potential for a framework to organize coastal referrals to address these barriers. Finally, this study demonstrated that GPs are interested in being involved in future research on the effects of the coast on health. The discussion mainly highlighted the patient specificity of adherence to and effectivity of coastal prescriptions, and framed this in comparison with green and medication prescription. The limitations were the limited sample size, selection bias, and focus on coastal visits and not on virtual alternatives to coastal visits. To address these issues, future research should capture the perspective of a representative nation-wide sample of GPs on coastal prescription. Also, fundamental and applied research is warranted on the effectivity and applicability of coastal vs. green vs. medication prescriptions.

## Data Availability

The original contributions presented in this study are included in the article. Further inquiries can be directed to the corresponding author.

## Supplementary Materials

The following supporting information can be downloaded at https://www.mdpi.com/article/10.3390/healthcare13131599/s1, Supplementary Material S1: Semi-structured interview guide.

## Abbreviations

The following abbreviations are used in this manuscript:

GP	General practitioner
SES	Socio-economic status
