# Supplementary material for: General Practitioners’ Perceptions on Prescribing Coastal Visits for Mental Health in Flanders (Belgium)
Source: Healthcare (Basel). 2025 Jul 3;13(13):1599. doi: 10.3390/healthcare13131599 (PMC12250064; doi:10.3390/healthcare13131599)
Supplement: Supplementary file 1 [file healthcare-13-01599-s001.zip › healthcare-3698403-supplementary.pdf]

# [English/ENG translation] Semi-structured interview guide

<u>Greeting</u>:

Good morning/afternoon [Name of healthcare provider]. Thank you for being willing to answer a few questions.

I will first reframe the purpose of this research. There is increasing evidence that a visit to the coast may promote mental health, and in the future, it might be a good idea to prescribe coastal visits to patients. Our team wants to explore this further next year. As part of my master's thesis, I am first attempting to map out how general practitioners feel about prescribing coastal visits. Specifically, I am interested in the attitude of GPs toward prescribing or recommending a coastal visit.

The conversation will begin with a few questions about your demographic background. Then, I will ask about your personal experience with “prescribing the coast” within your practice. After that, I will inquire about your perspective on the organizational and societal challenges of prescribing a coastal visit, and I will conclude with a few research-related questions. I would like to thank you again for participating in our research.

I would also like to remind you that, as a participant in this study, you are free to stop the interview at any time. If there are any questions you would prefer not to answer, you are not obligated to do so. Additionally, before we start the recording, may I ask you to read and sign the informed consent document? If you agree, this interview will be recorded, but your responses will remain anonymous, and the recording will not be shared with any external parties.

**Do you have any questions so far?** If not, we can begin. If that’s okay with you, I will now start the recording.

<u>Part 1A: Basic information about the general practitioner being interviewed</u>:

- **How many years have you been working as a GP?**
- **How old are you, and what is your date of birth?**
- **What is the postal code of your practice?**

<u>Part 1B: Experience Related to the Coast:</u>

- **What is your personal experience with the coast?**
- **How would you describe your relationship with the sea and coastal environment?**
- **How does this translate into activities in your life? For example, is the sea your laptop background?**
- **How often do you go to the coast?**
- **What do you think are the effects of the coast on an individual, both physically and mentally?**

Part 2: Previous Recommendations:

**Have you ever recommended a visit to the coast (or other nature) as part of a mental health treatment plan for a patient?**

*If yes:*

- **How often do you generally recommend a visit to the coast or nature?**
  - **For which types of patients, i.e., with which symptoms?**
- **How did you present this referral or recommendation to the patient?**
- **What factors played a role in your willingness to recommend a coastal or nature visit?**
- **How effective do you think a coastal visit is in combating poor mental health? Why do you think so?**
- **Do you need more evidence to recommend a coastal visit?**
  - **If so, what kind of research or evidence would you need to build that confidence?**
- **Are you aware of other doctors/colleagues who also recommend coastal or nature visits?**

*I will summarize what the doctor tells me and ask if they have anything to add.*

*If No:*

- **Was your choice not to recommend a coastal or nature visit a conscious one?**
- **What factors played a role in your decision not to recommend a coastal or nature visit?**
- **Do you think there are specific groups of patients for whom a coastal or nature visit could be beneficial?**
- **How effective do you think a coastal visit is in combating poor mental health? Why do you think so?**
- **Do you need more evidence to recommend a coastal visit?**
  - **If so, what kind of research or evidence would you need to build that confidence?**
- **Are you aware of other doctors/colleagues who do recommend a coastal visit?**

*I will summarize what the doctor tells me and ask if they have anything to add.*

Patient experience and feedback:

**Can you share any stories or feedback from patients regarding the recommendation of coastal or nature visits for improving mental health?**

Part 3: Challenges and Limitations:

We already know that people feel healthier by the coast, and it's possible that visiting the coast more frequently could prevent and help treat many mental health issues. In the future, general

practitioners may, for instance, be encouraged to prescribe coastal visits to patients, similar to prescribing physical activity.

What do you think are the possible challenges or limitations in recommending more frequent coastal visits for mental health within your practice and with your patients? Points to discuss, if they do not come up, explicitly ask about:

**What do you think are the possible challenges or limitations in recommending more frequent coastal visits for mental health within your practice and with your patients?**

- *Points to discuss, if they do not come up, explicitly ask about:*
- *Doctor-patient communication*
- *Follow-up of the patient*
- *Travel and location-related costs*
- *Accessibility of the coast*
- *Patient preferences*
- *Doctor and patient knowledge about the benefits of coastal visits*

Now, I would like to discuss the challenges and limitations at a societal level.

**What do you think are the possible challenges or limitations in recommending such activities for mental health on a societal level?**

*Sub-questions to ask if not addressed:*

- Collaboration with specialists: Would you like to see collaboration with other healthcare providers or specialists when recommending coastal visits as part of a treatment plan?
    - If yes, can you describe these collaborations?
    - Would you like more specialists to be involved?
- *Top-down guidelines*: In your opinion, is there a need for a framework, for example from the Flemish or federal government, to offer coastal prescriptions to patients? Do you see possibilities for financial or other support from health insurance funds or organizations (such as Logo's or any others you can think of) to facilitate coastal prescriptions in practice?
- *Bottom-up guidelines*: Do you think there should be greater patient awareness about the benefits of visiting the coast?
- *Healthcare system and insurance (societal level)*: What role do you think insurance companies and healthcare systems should play in supporting this type of therapy?

Part 4: Involvement in research

Finally, I would like to ask you some questions to connect this interview with our research. We are currently considering studying the effects of the coast on mental health in a more realistic context. Previously, we always worked with healthy study populations, but now we want to measure the

effects of the coast on patients who visit their GP with complaints that may result from reduced mental health (e.g., stress, tension headaches, hyperventilation). As researchers, we would follow these patients for several weeks and conduct assessments through questionnaires, brain scans, and physiological measurements (e.g., of the nervous system, such as vagus nerve activity and the sympathetic nervous system) while they visit the coast with a certain frequency. To reach these patients, we would ask general practitioners to refer them to us, the researchers.

**I'll give a concrete example of how we envision this in practice, but first, we are interested in how much you think GPs should be involved in the different stages of this research, such as in defining the research questions, conducting the measurements, developing the methodology, patient follow-up, and interpreting the results.**

One specific idea is to create a flyer with a brief summary of all relevant information about the potential effects and what the study entails, which the GP could give to a patient during or after a consultation, so the patient receives all the necessary information about the project efficiently. **Would you be inclined to do it this way, or how would you approach it differently?**

Lastly, we also have questions about the "dose" of coastal visits. We know that people who live closer to the coast visit it more often. Additionally, every patient's personal situation and available time differ. To investigate dose-response relationships, we as researchers would want both people living near the coast and further inland, with both flexible and busy schedules, to visit the coast with both low and high frequency. **What are your thoughts on this? Would you be more inclined to let patients choose the frequency themselves, or would you prescribe it, and why? Please consider this question without regard to the financial aspect. What frequencies would you recommend to your patients? What is the lowest and highest frequency you would suggest in the context of research? (Keep in mind your answers about practical feasibility and expected effects of coastal visits).**

<u>*Closing:*</u> We've reached the end of our interview.

**Is there anything else you would like to add? Finally, I'd like to ask if you would like to stay informed via email about the results of this research?**

***Over the coming years, several studies will be initiated in which we will rely on general practitioners. The results of this interview will certainly be helpful in optimizing the methodologies of these studies. May we add your email address to our list of interested parties so that you can receive an invitation to participate in these studies?***

*Thank you very much for your participation; we greatly appreciate it. Your insights are very valuable to the progress of our research. You will be kept informed of the results via email.*

# [Dutch/NL] Semi-gestructureerd interview

Begroeting:

Goedemorgen/goedemiddag [Naam hulpverlener]. Bedankt dat u bereid bent om enkele vragen te beantwoorden.

Ik zal eerst opnieuw kaderen waar het onderzoek over gaat. Er is steeds meer evidentie dat een bezoek aan de kust bevorderend zou zijn voor de mentale gezondheid, en in de toekomst is het misschien een goed idee om patiënten op doktersvoorschrift naar de kust te sturen. Ons team wil dat volgend jaar verder onderzoeken. In het kader van mijn masterproef wordt getracht eerst in kaart te brengen hoe huisartsen tegenover de kust op voorschrift staan. Specifiek ben ik geïnteresseerd in de attitude van de huisarts tegenover het voorschrijven of aanbevelen van een kustbezoek.

Het gesprek zal starten met enkele vragen over uw demografische achtergrond. Daarna zal ik uw persoonlijke ervaring met 'de kust op voorschrift' binnen uw praktijk bevragen. Daarna bevraag ik uw visie op de organisatorische en maatschappelijke uitdagingen van een kustbezoek op voorschrift en ik sluit af met enkele onderzoek gerelateerde vragen. Bij deze wil ik u dus zeker bedanken om mee te doen aan ons onderzoek.

Ik wil u er graag aan herinneren dat u als deelnemer van deze studie vrij bent om te stoppen met het onderzoek wanneer u dit zou willen. Mochten er vragen zijn die u liever niet wil beantwoorden dan bent u niet verplicht om een antwoord te geven. Ook wil ik u voor de start van de opname vragen om het document voor geïnformeerde toestemming door te lezen en te handtekenen? Indien u hiermee instemt zal dit interview opgenomen worden maar uw antwoorden blijven anoniem en de opname zal ook niet gedeeld worden met externen.

**Hebt u tot nu toe al vragen?** Zo niet, dan kunnen we beginnen. Als dit goed is voor u zal ik vanaf nu dan ook de opname starten.

Basisinformatie over de arts:

- **Aantal jaren dat u werkt als huisarts?**
- **Hoe oud bent u en wat is uw geboortedatum?**
- **Wat is de postcode van uw praktijk?**

Ervaring met betrekking tot de kust:

**Wat is uw eigen ervaring met de kust?**

- **Hoe zou u uw relatie met de zee en kustomgeving omschrijven?**
- **Hoe vertaalt zich dit in de activiteiten in uw leven? Bv: De achtergrond van uw laptop is de zee**

- **Hoe vaak gaat u naar de kust?**
- **Wat denkt u dat de effecten zijn van de kust op een individu, zowel fysiek als mentaal?**

Eerdere aanbevelingen:

**Heeft u ooit een bezoek aan de kust en/of natuur aanbevolen als onderdeel van een behandelplan voor de mentale gezondheid van een patiënt?**

*Zo ja:*

- **Hoe vaak beveelt u doorgaans een bezoek aan de kust of natuur voor?**
    - **Voor welk type patiënten, i.e. met welke symptomen?**
- **Hoe hebt u deze doorverwijzing of aanbeveling bij de patiënt aangebracht?**
- **Welke factoren hebben een rol gespeeld in uw bereidwilligheid om een kustbezoek of natuurbezoek aan te bevelen?**
- **Hoe effectief is een kustbezoek volgens u om een verminderde mentale gezondheid tegen te gaan? Waarom denkt u daar zo over?**
- **Hebt u meer evidentie nodig om een kustbezoek aan te bevelen?**
    - **Zo ja, wat voor onderzoek of evidentie zou u nodig hebben om dat vertrouwen het meest aan te wakkeren?**
- **Bent u zich bewust van andere dokters/collega's die ook een kust -of natuurbezoek aanbevelen?**

*Ik vat samen wat de huisarts mij vertelt en vraag of hij/zij hier nog iets aan toe te voegen heeft.*

*Zo nee:*

- **Was het niet aanbevelen van een kust -of natuurbezoek een bewuste keuze?**
- **Welke factoren hebben een rol gespeeld in uw keuze om een kust -of natuurbezoek niet aan te bevelen?**
- **Denkt u dat er bepaalde patiëntengroepen zijn waarbij een kust -of natuurbezoek nuttig zou kunnen zijn?**
- **Hoe effectief is een kustbezoek volgens u om een verminderde mentale gezondheid tegen te gaan? Waarom denkt u daar zo over?**
- **Hebt u meer evidentie nodig om een kustbezoek aan te bevelen?**
    - **Zo ja, wat voor onderzoek of evidentie zou u nodig hebben om dat vertrouwen het meest aan te wakkeren?**
- **Bent u zich bewust van andere dokters/collega's die wel een kustbezoek aanbevelen?**

*Ik vat samen wat de huisarts mij vertelt en vraag of hij/zij hier nog iets aan toe te voegen heeft.*

Ervaring van de patiënten en feedback van patiënten:

**Kunt u verhalen of feedback van patiënten delen met betrekking tot het aanbevelen van kust -of natuurbezoeken voor verbetering van de mentale gezondheid?**

Uitdagingen en beperkingen:

We weten reeds dat mensen zich gezonder voelen aan de kust, en het zou kunnen dat het vaker bezoeken van de kust heel wat mentale problemen kan voorkomen en helpen genezen. In de toekomst zou men bijvoorbeeld huisartsen kunnen aanbevelen om kustbezoeken voor te schrijven aan de patiënt, gelijkaardig aan 'bewegen op verwijzen'.

**Wat zijn volgens u mogelijke uitdagingen of beperkingen bij het aanbevelen van het vaker bezoeken van de kust voor de mentale gezondheid in uw praktijk en bij uw patiënten?**

*Puntjes die aan bod kunnen komen, indien ze niet aan bod komen, er expliciet naar vragen: Communicatie van dokter naar patiënt toe*

- *Opvolging van de patiënt*
- *Kosten m.b.t reizen en locatie*
- *Toegankelijkheid van de kust*
- *Voorkeuren van de patiënt*
- *Kennis over de voordelen van een kustbezoek van de dokter en van de patiënt*

Dan zou ik nu graag de uitdagingen en beperkingen op maatschappelijk niveau bespreken.

**Wat zijn volgens u mogelijke uitdagingen of beperkingen bij het aanbevelen van dergelijke activiteiten voor de mentale gezondheid op maatschappelijk niveau?**

*Subvragen die bevraagd kunnen worden als ze niet aan bod komen:*

- *Samenwerking met specialisten: Zou u graag hebben dat een samenwerking mogelijk is met andere zorgverleners of specialisten wanneer je kustbezoeken voorstelt als een onderdeel van de behandeling?*
    - *Zo ja, kunt u deze samenwerkingen beschrijven?*
    - *Zou u graag hebben dat er meer specialisten bij betrokken zijn?*
- *Top down richtlijnen: Is er volgens u nood aan een kader, bijvoorbeeld vanuit de Vlaamse of federale overheid, om kustvoorschriften aan te bieden aan patiënten? Ziet u mogelijkheden vanuit de ziekenkas of Logo's (of andere instanties waar u aan denkt) om financieel of op andere manier een kustvoorschrift in de praktijk te faciliteren?*
- *Bottom-up richtlijnen: Vindt u dat er een grotere bewustwording moet zijn onder de patiënten over de voordelen van een bezoek aan de kust?*
- *Zorgsysteem en verzekering (maatschappelijk niveau): Wat is naar uw mening de rol van verzekeringsmaatschappijen en gezondheidszorgsystemen bij het ondersteunen van dit soort therapieën?*

Ten slotte wil ik u nog enkele vragen stellen om het voorgaande interview te koppelen aan ons onderzoek. We zijn momenteel aan het denken om de effecten van de kust op mentale gezondheid te

onderzoeken in een realistischere context. Voorheen werkten we altijd met gezonde studiepopulaties, maar nu wensen we de effecten van de kust te meten van patiënten die bij de huisarts komen met klachten die het gevolg kunnen zijn van een verminderde mentale gezondheid (vb. stress, spanningshoofdpijn, hyperventilatie). Wij als onderzoekers zouden die patiënten gedurende enkele weken opvolgen, en metingen doen via vragenlijsten, hersenscans, en fysiologische metingen (e.g. van het zenuwstelsel: e.g. activiteit van de nervus vagus, sympathische zenuwstelsel), terwijl ze met een bepaalde frequentie de kust bezoeken. Om die patiënten te bereiken zouden we huisartsen vragen om deze patiënten door te sturen naar ons, de onderzoekers.

**Ik geef meteen een concreet voorbeeld van hoe we dat in de praktijk zien, maar eerst zijn we geïnteresseerd in hoeverre u denkt dat huisartsen betrokken moeten zijn bij de verschillende stadia van dit onderzoek: i.e. bij het definiëren van de onderzoeksvragen, de uit te voeren metingen, het uitwerken van de methodologie, de opvolging van de patiënt, en interpretatie van de resultaten?**

Een concreet idee is om een flyer op te stellen met in het kort alle relevante informatie omtrent over wat de mogelijke effecten zijn en wat het onderzoek inhoudt. en dat de huisarts deze flyer zou kunnen aanreiken aan een patiënt tijdens of na een consultatie, zodanig dat deze op een efficiënte manier alle nodige informatie ontvangt over het project. **Zou u geneigd zijn dit zo te doen, of hoe zou u het anders aanpakken?**

Ten slotte stellen we ons ook vragen bij de dosis kustbezoeken. We weten dat mensen die dichter bij de kust wonen de kust vaker bezoeken. Daarnaast is de persoonlijke situatie en beschikbare tijd voor elke patiënt verschillend. Om dosis-response relaties na te gaan wensen wij als onderzoekers dat zowel mensen als aan de kust als meer naar het binnenland, en zowel met een flexibele als drukke planning zowel met een lage als hoge frequentie de kust bezoeken.

**Hoe denkt u daarover? Zou u eerder geneigd zijn om de patiënten zelf over de frequentie te laten kiezen, of zou u het eerder opleggen en waarom? Deze vraag mag bekeken worden onafgezien van het financiële aspect.**

**En welke frequenties zou u aanraden aan uw patiënten, wat is de laagste en hoogste frequentie die u zou aanbevelen in de context van onderzoek? (Houd hierbij de reeds beantwoorde vragen op vlak van praktische haalbaarheid en verwachte effecten van een kustbezoek in het achterhoofd).**

*Afsluiting:* We zijn aan het einde van ons interview gekomen.

**Is er nog iets dat u zou willen toevoegen? Tenslotte wou ik u nog vragen of u graag verder op de hoogte wordt gehouden via email over de resultaten van dit onderzoek?**

**Er zullen in de komende jaren verschillende studies geïnitieerd worden waarbij we beroep zullen doen op huisartsen De resultaten van dit interview zullen alvast nuttig zijn om de methodieken van deze studies te optimaliseren. Mogen wij uw emailadres op onze lijst van geïnteresseerden plaatsen, zodat u een uitnodiging kan ontvangen om deel te nemen aan deze studies?**

Heel erg bedankt voor uw deelname, we stellen het erg op prijs. Uw inzichten zijn zeer waardevol voor de vooruitgang van ons onderzoek. U zal op de hoogte worden gehouden van de resultaten via mail.
